# Limited Term Appointments of Medical Officers of Health

**Published:** 1907-08-24

**Authors:** 


### Public Health and Hygiene.

### LIMITED TERM APPOINTMENTS OF MEDICAL OFFICERS OF HEALTH.

At a recent meeting of the Rural District Council of the New Forest, the Clerk intimated the receipt of a communication from the Local Government Board announcing the expiration of the term of the appointment of Dr. Sheppard as Medical Officer of Health. The occasion was seized upon by a few of the Councillors as a timely one for "applying the screw. We quote from the *Hampshire Independent*:—

The Rev. J. J. Daly urged that in future before the medical officer reported to the Local Government Board he should submit his report to the Council. In Lyndhurst they thought they were not fairly treated in the last report, and when he came to the Council and found it had been forwarded to the Local Government Board without being submitted to the Council he thought it was a most extraordinary proceeding. In order that that might not happen again he would propose that in future the medical officer should submit his annual report to the Council before forwarding it to the Local Government Board. The medical officer must have known that they were doing their best to supply Emery Down with water, *and he thought he should not have referred to it in his report*.—Mr. Fletcher proposed the re-election of Dr. Sheppard at his former salary, subject to the approval of the Local Government Board.—Mr. Lermitte seconded the proposition.—The Rev. J. J. Daly proposed, as an amendment, that the appointment be deferred till the next Board day, to give Dr. Sheppard an opportunity of saying whether he would acquiesce in the request of the Council.—Mr. Wadsworth seconded the amendment, and suggested that other candidates might be invited to apply. ("No, no.").—The Rev. J. J. Daly said he would withdraw the amendment, and the proposition was carried.

The Councillors who submitted the Medical Officer of Health to the indignity of the proceedings reported doubtless felt that they had a legitimate grievance, but it is intolerable that an officer who is carrying out the explicit instructions of his official superiors should be placed in so ignominious a position through the ignorance of the Councillors whom he more immediately serves.

A general order of the Local Government Board as to the duties of medical officers of health provides that: "He *shall* give immediate information *to us* of any outbreak of dangerous epidemic disease within the district, and *shall* transmit *to us* a copy of each annual report and of any special report. He *shall* make a special report *to us* of the grounds of any advice which he may give to the sanitary authority with a view to their requiring the closure of any school or schools," etc.

Other officers of local authorities whose duties do not require them to report to the superior authority are not, as the medical officer is, appointed from year to year. Why should this distinction be made in the case of the Medical Officer of Health? It would be a simple act of justice if the Local Government Board would, before approving the conditions of appointment of a medical officer of health, insist that the term for which he is appointed shall be indefinite, as is the case with other municipal officers.

